# Effects of Prolonged Cryogenic Exposure on the Electrical Degradation of Stator Main Insulation in Wind Turbines

**DOI:** 10.3390/ma19091675

**Published:** 2026-04-22

**Authors:** Zheng Dong, Haitao Hu, Junguo Gao, Mingpeng He, Zhongyi Huang, Yanli Liu

**Affiliations:** 1 School of Electrical and Electronic Engineering, Harbin University of Science and Technology, Harbin 150080, China; dongzheng0712@163.com (Z.D.); huhaitaodianqi@hrbust.edu.cn (H.H.); huangzhongyi333@163.com (Z.H.); liuyanli@hrbust.edu.cn (Y.L.); 2 State Key laboratory of Electrical Insulation and Power Equipment, Xi’an Jiaotong University, Xi’an 710049, China; 3 Dongfang Electric Machinery Co., Ltd., Deyang 618000, China; ligong038@126.com

**Keywords:** low temperature, microcrack effect, electrical performance

## Abstract

Epoxy-glass-mica composite materials are widely used as electrical insulating materials in high-voltage rotating machinery due to their layered structure and excellent dielectric properties. Taking the F-class epoxy glass with a small amount of rubber powder mica tape commonly used as the main insulation of wind turbine stator coils as the research object, 7-day, 14-day, 21-day, and 28-day low-temperature treatment tests were conducted at −50 °C. The surface morphology and chemical structure changes of the materials were characterized by SEM and FTIR, and the influence laws of low-temperature treatment on the electrical properties of the mica tape insulation materials were systematically studied. The experimental results show that the low-temperature environment will induce microcracks and interface delamination and other structural damages, but no obvious change in the chemical structure of the mica tape was observed. With the extension of the low-temperature treatment time, the electrical properties of the mica tape show a deteriorating trend, and after 28 days of low-temperature treatment, the breakdown field strength of the F-class mica tape decreased by approximately 18.5%, and the volume conductivity overall increased by about two orders of magnitude. This indicates that the microcrack defects induced by low-temperature will lead to an enhanced electrical-thermal coupling effect in the insulation structure, thereby accelerating the degradation process of the insulation material. This reveals the degradation mechanism of wind turbine stator main insulation from “structural damage” to “performance degradation” and then to “insulation aging” under low-temperature conditions, providing a theoretical basis for the design and reliability assessment of insulation systems in wind turbine generators in cold regions.

## 1. Introduction

With the advancement of the Belt and Road Initiative, major domestic power generation equipment manufacturers have undertaken unit installation, maintenance, and retrofitting projects in frigid regions such as Central Asia and Northern Europe. The primary insulation of wind turbine stator windings is subjected to multiple thermal, mechanical, and environmental stresses during both manufacturing and operation. These stresses accelerate insulation aging, thereby compromising the reliability and service life of the motor. Consequently, the lifespan of the primary insulation often determines the overall operational longevity of the entire unit [1,2]. The performance of wind turbine main insulation is determined by the insulation system. The main insulation materials constituting this system [3] include impregnating resin and the corresponding mica tape system [4]. Mica tape is typically composed of mica paper, epoxy resin, and reinforcing materials [5]. Since the 20th century, it has been the dominant key material in the insulation structure of motor stator windings [6], and it should possess good electrical characteristics and mechanical properties sufficient to ensure the long-term stable operation of the power system [7]. The commonly used mica tape insulation systems in current engineering mainly include two grades: F grade and H grade. Wind turbines generally adopt insulation systems with a heat resistance level of F grade. According to several major wind power markets in China, the mica tapes used in conjunction with these insulation systems are mostly F-grade epoxy resin mica tapes.

Currently, extensive research has been conducted by scholars worldwide on high- and low-temperature operation, electrical performance optimization, and the impact of actual operating environments. Studies have found that composite materials undergo significant changes at low temperatures, with their electrical and physical properties notably altered, including changes in dielectric response, conductivity, and partial discharge behavior, highlighting the necessity for systematic research on insulating composites at low temperatures [8,9,10]. Jiang et al. found that the DC breakdown strength of epoxy resin is higher at low temperatures than at room temperature [11]. J. Gerhold et al. discovered that the dielectric loss of epoxy resin decreases as temperature decreases [12]. Liangliang Q et al. characterized the thermal expansion coefficient (CTE) and tensile properties within the temperature range of 300 K to 90 K, indicating that the elastic modulus and tensile strength increase linearly with the decrease in temperature [13]. Existing studies generally indicate that the short-term electrical performance of polymeric insulating materials may improve under low-temperature conditions, manifested by reduced conductivity, suppressed dendritic growth, and enhanced breakdown strength. However, such results are predominantly obtained from short-duration tests and therefore cannot adequately represent the long-term service behavior of insulation systems subjected to prolonged cryogenic exposure. Zhou Yuanxiang and others aimed to investigate the impact of low-temperature environment on the electrical properties of epoxy resin. They conducted low-temperature treatment tests on the epoxy resin at −20, −40, and −60 °C for 7, 15, and 30 days, respectively. As the duration of low-temperature treatment increased, micro-cracks would appear on the surface of the epoxy resin and its deterioration would continue. Meanwhile, the DC breakdown strength and resistivity of the epoxy resin showed a trend of first increasing and then decreasing. Moreover, this changing trend became more obvious as the treatment temperature decreased [14]. Sankar et al. observed microcracks forming on the surface of epoxy resins exposed to prolonged low-temperature environments [15]. indicating that low temperatures induce structural damage within the material, potentially weakening its macroscopic electrical properties. Research on long-term low-temperature conditions has revealed that such environments may alter the internal space charge distribution and local discharge behavior of materials, thereby accelerating the degradation process of insulating materials. Moreover, the decline in the mechanical properties of materials under low-temperature conditions is also one of the important factors leading to insulation failure. The study found that the insulation failure form of polymers under low temperature is the expansion of material cracks resulting in insulation failure [16,17,18]. Z. B. Zhang et al. studied the degradation mechanisms of epoxy glass fiber reinforced plastic at 278.7 K and 223 K for 53 h and at 153 K for 30 h under low temperature conditions. They found that the chemical corrosion of aging products and the discharge energy had little effect on the deterioration of the material. The degradation mechanism of epoxy glass fiber reinforced plastic at low temperatures can be attributed to the combined effect of space charge accumulation and mechanical property degradation [19]. Z. H. Zhang and others studied the high-temperature insulation performance of mica belts and constructed a hexagonal boron nitride (h-BN) thermal conductivity network, providing new ideas for high thermal conductivity insulating materials and structures [20]. Dietmar Lenko employed non-destructive testing techniques and thermal mechanical characterization methods to characterize typical faults and defects in the winding insulation, as well as the anti-decomposition ability, in order to further assess the reliability of the stator insulation for high-voltage rotating machines [21]. Although scholars have conducted systematic studies on resin systems for motor insulation [22] and the compatibility between mica tape and impregnating varnish [4], systematic experimental data regarding the breakdown performance and changes in dielectric properties of stator main insulation in wind turbines under long-term low-temperature storage or operational conditions are still lacking. However, most existing studies focus on the short-term electrical performance measured directly under low-temperature conditions, and the evolution of electrical performance after long-term low-temperature exposure as well as subsequent room temperature testing is still not well understood. In particular, the coupling between microstructural damage caused by low temperatures in epoxy-mica stator insulation and electrical performance degradation has not been systematically elucidated.

Therefore, this paper selects a typical mica tape system for the stator main insulation of wind turbines as its research subject. By subjecting the material to long-term low-temperature treatment and conducting electrical performance tests at room temperature, it systematically analyzes the influence patterns of low-temperature environments on key electrical properties such as the breakdown field strength of insulation materials. The research findings provide experimental evidence and theoretical support for assessing the insulation reliability of wind turbines during long-term operation in frigid regions or under low-temperature storage conditions.

## 2. Materials and Methods

### 2.1. Low-Temperature Treatment

At present, the wind power insulation system being researched by Dongfang Electric Group Co., Ltd. has the most stringent minimum temperature requirements. Specifically, the minimum operating temperature is −40 °C, and the minimum tolerance temperature is −50 °C. Since generators generate a large amount of heat during operation, the minimum tolerance temperature is a more critical parameter in storage and logistics design. The cold cycle test primarily simulates the effects of high and low temperature shocks on the main insulation of motor stator windings during transportation and storage due to extreme environmental conditions [23]. In accordance with GB/T 2423.1 “Environmental Testing for Electrical and Electronic Products—Part 2: Test Methods—Test A: Low Temperature” [24], conduct low-temperature endurance test Ab for the minimum survival temperature. Each cycle shall have a holding time of 168 h, with a total of 4 cycles required. The selected low-temperature exposure protocol is designed to simulate the conditions of long-term storage and transportation of wind turbines in cold regions, as the insulation system may experience prolonged sub-zero environments before being put into service.

### 2.2. Test Samples

Low-adhesive mica tape, characterized by its reduced adhesive content, has emerged as the fastest-growing product in the past decade [25]. Extensive practical experience and achievements in its application within electric motors have been documented both domestically and internationally [26]. The samples used in the low-temperature testing for this paper are Class F stator coils. The samples for both macro- and micro-level testing are the primary insulation material of the coil structure-Epoxy Glass Clad Mica Tape. The specific composition is detailed in Table 1.

After low-temperature treatment, thin sections were cut from the region near the coil slots. The insulation layers, from outer to inner, consisted of slot insulation, glass tape, and mica tape. The mica tape was removed for testing. Prior to low-temperature treatment, samples at 7 days, 14 days, 21 days, and 28 days were recorded as: 0 d, 7 d, 14 d, 21 d and 28 d.

### 2.3. Testing Method

The SU8020-SEM model equipment manufactured by Hitachi High-Technologies Corporation of Tokyo, Japan was employed to characterize the microstructure of the filler and its dispersion within the medium. Prior to testing, a conductive gold film was deposited onto the sample surface to prevent charge accumulation over time, thereby enhancing the clarity and accuracy of the SEM analysis. The gold sputtering was performed at a current of 50 mA for 80 s, followed by surface imaging.

The primary objective of this experiment was to identify the types of functional groups and whether reactions have occurred between molecular functional groups. The Fourier-transform infrared spectroscopy (FTIR) data in this paper was obtained using an EQUINOX-55 FTIR spectrometer (Thermo Fisher Scientific, Waltham, MA, USA). The film size used for testing was 4 cm × 3 cm, with all tests conducted at room temperature. The measurement range was set from 500 cm^−1^ to 4000 cm^−1^.

The DC conductivity of the sample was measured at different temperatures using a three-electrode system. The DW-P153-5ACF3 high-voltage power supply from ADC (Harrison, AR, USA) provided the voltage, the ZC36 high-resistance meter (Shanghai Suhai Electric, Shanghai, China) measured the current through the sample, and the GP/GDW150 high-low temperature test chamber (Shanghai Guangpin Test Equipment Manufacturing Co., Ltd., Shanghai, China) controlled the temperature. This setup enabled conductivity measurements at various temperatures. After stabilization at ambient temperature for 30 min, the voltage on the DC power supply instrument was adjusted to 1.4 kV. Once the current reached a steady state, the reading was recorded and the temperature was increased to the next value. This process was repeated.

The dielectric property tester uses the Alpha-A type wideband dielectric spectrum analyzer produced by Novocontrol, Montabaur, Germany. The test sample size is 12 mm × 12 mm, with a sample thickness of 100 μm. After depositing circular aluminum film electrodes with a diameter of 9 mm on both sides, the prepared samples are placed in a vacuum drying oven for a 24 h short-circuit discharge treatment. The measurement temperature range is −30 °C to 140 °C, and the measurement frequency is 50 Hz.

DC breakdown strength testing was conducted using Huabo Technology’s HJC-100 kV breakdown tester (Suzhou, China) with a cylindrical electrode system measuring 25 mm diameter × 75 mm diameter. The voltage rise rate was controlled at 1 kV/s during testing. The specimen thickness was approximately (0.5 ± 0.05) mm, and tests were conducted at room temperature. To prevent surface flashover, specimens and electrodes were immersed in transformer oil. The diameter of the plate electrode used in the breakdown experiment is set at 50 mm, and the thickness is 5 mm. The test adopts a rapid pressurization method, applying a sinusoidal voltage, starting from 0 kV and increasing the voltage at a rate of 2 kV/s until the sample breaks down. Each sample group underwent 10 repeated tests, followed by Weibull analysis.

## 3. Results Microstructural Characterization

### 3.1. Microstructural Characterization

#### 3.1.1. Scanning Electron Microscopy of Insulating Materials

**Figure 1:**
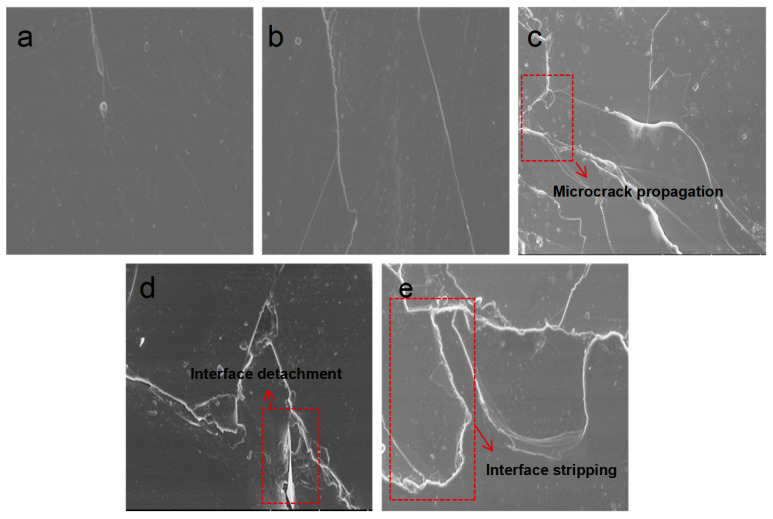
SEM images of Class F insulation materials after different cryogenic treatment durations. (**a**) SEM image of Class F insulating material prior to low-temperature treatment. (**b**) SEM image of Class F insulation material after 7 days of low-temperature treatment. (**c**) SEM image of Class F insulation material after 14 days of low-temperature treatment. (**d**) SEM image of Class F insulation material after 21 days of low-temperature treatment. (**e**) SEM image of Class F insulation material after 28 days of low-temperature treatment.

Figure 1 shows scanning electron microscope (SEM) images of the primary insulation materials of the stator after different cryogenic exposure cycles. Figure 1a shows the insulating material before low-temperature treatment, exhibiting a smooth surface without cracks. After 7 days of treatment at −50 °C, fine cracks were observed on the surface of the F-grade material, as depicted in Figure 1b. After 14 days, the cracks in the F-grade material widened and propagated, forming a locally interconnected crack network and exhibiting phenomena resembling brittle interlaminar delamination, as shown in Figure 1c. After 21 days, the crack density in the F-grade material increased significantly, with multiple cracks penetrating the surface and forming localized spalling pits, as depicted in Figure 1d. After 28 days of low-temperature exposure, extensive damage appeared in the F-grade material, including wide and deep cracks along with interfacial delamination, as shown in Figure 1e. SEM observations revealed the progressive evolution of low-temperature damage, from microcrack nucleation to crack coalescence and interfacial separation. This behavior resulted from severe thermal expansion mismatch between the epoxy matrix and the mica-glass reinforcement. Under prolonged exposure, crack interconnection and interfacial failure became dominant, leading to irreversible structural degradation and providing preferential pathways for electrical failure. However, no filler precipitation was observed in the SEM images, indicating that the aging process did not cause molecular chain breakage or macromolecular decomposition in the matrix. The damage mechanism was primarily mechanical failure, manifested as cracking and delamination, rather than chemical degradation.

#### 3.1.2. Fourier-Transform Infrared Spectroscopy of Insulating Materials

**Figure 2:**
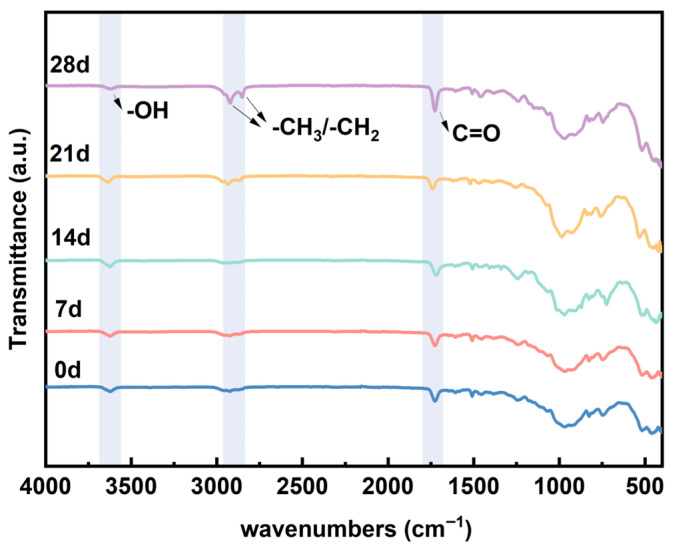
Infrared spectra of Class F insulation materials after different cryogenic treatment durations.

Infrared spectroscopy investigates the molecular structure of insulating materials and identifies compounds through qualitative analysis [27]. Infrared spectroscopy is a powerful technique for identifying functional groups present in materials by measuring the absorption of infrared radiation at specific wavelengths. Different bonds vibrate at characteristic frequencies, producing unique absorption bands. Figure 2 shows the infrared spectra of epoxy glass-reinforced mica tape after 7, 14, 21, and 28 days at −50 °C. The absorption peak at 3633 cm^−1^ is attributed to the stretching vibration absorption peak of hydroxyl (-OH) groups [28]. which may originate from hydroxyl groups in the polymer backbone, terminal groups, or adsorbed moisture. The peak at 2921 cm^−1^ corresponds to the stretching vibration of the -CH_2_ group, while the absorption peak at 1730 cm^−1^ originates from the carboxyl group. Peaks below 1500 cm^−1^ comprise complex absorption bands resulting from various bending and stretching vibrations of C-O, C-C, and C-H bonds. The infrared spectrum indicates that after low-temperature treatment, the broad peak of -OH in the spectrum gradually intensifies, and at the same time, the absorption band related to the carboxyl group also shows an increasing trend over time. The stretching vibration peaks of -CH_3_/-CH_2_ change relatively little. The analysis suggests that the microcracks or microporous structures caused by low temperature make the surface more prone to adsorbing trace moisture in the environment and forming hydrogen bond associations, thus resulting in an enhanced -OH absorption; on the other hand, the generation of microdefects and interface relaxation will increase the exposure degree of local active sites, causing slight oxidation-related carboxyl absorption to enhance on the surface. The main influence of low temperature on the two insulating materials is not the shift in peak positions, but the change in peak intensity, mainly due to the changes in surface polarity and interface state. The combined SEM and FTIR results confirm that cryogenic degradation of epoxy-mica insulation is dominated by mechanically induced damage, while the chemical backbone of the polymer matrix remains essentially intact. This distinction is critical for understanding the reversibility and long-term accumulation of low-temperature damage in insulation systems.

### 3.2. Electrical Properties

#### 3.2.1. Electrical Conductivity of Insulating Materials

**Figure 3:**
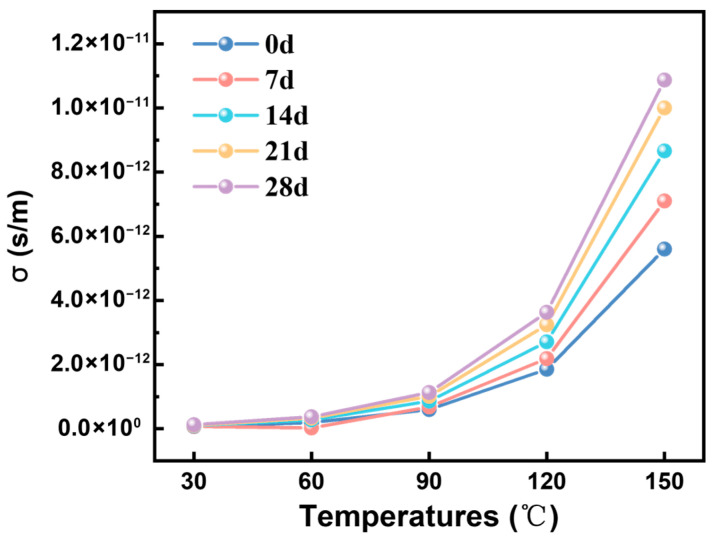
Electrical Conductivity of Class F Insulation Materials After Different Low-Temperature Treatment Durations.

The conductance current is a steady current flowing through the test specimen, reflecting the material’s insulating properties and the microscopic characteristics of carrier transport within polymers [28]. Figure 3 shows the conductivity changes of F-class insulation material after 7, 14, 21, and 28 days at −50 °C. For all exposure conditions, conductivity increases with the rise in test temperature, indicating a typical temperature-dependent behavior consistent with thermal activation of charge transport. This behavior can be described by the Arrhenius equation, indicating that conductivity is controlled by the inherent thermal activation of charge carriers and the accumulated structural damage during previous low-temperature exposures. All samples show a good linear relationship in ln(σ)−1/T, confirming the existence of thermal activation conduction behavior. The extracted activation energy (Ea) is in the range of 0.41–0.49 eV, also within the typical range of polymer insulation materials. In addition to the temperature effect, conductivity increases with the increase in exposure time at all temperatures. However, in this study, electrical measurements were conducted after the samples were restored to room temperature. Therefore, the increase in conductivity reflects the residual structural deterioration rather than the instantaneous inhibition of carrier transport. This deterioration is mainly attributed to microcracks and interface gaps caused by mismatched thermal contraction within the layered cloudier band structure, which can lead to the disruption of dielectric continuity and provide additional paths for charge transport. Moreover, the increased number of cracks leads to a greater quantity of internal interfaces within the material. Under the influence of an alternating electric field, the interface polarization effect intensifies, further elevating dielectric loss. Macroscopically, this manifests as an increase in conductivity.

#### 3.2.2. Dielectric Constant of Insulating Materials

**Figure 4:**
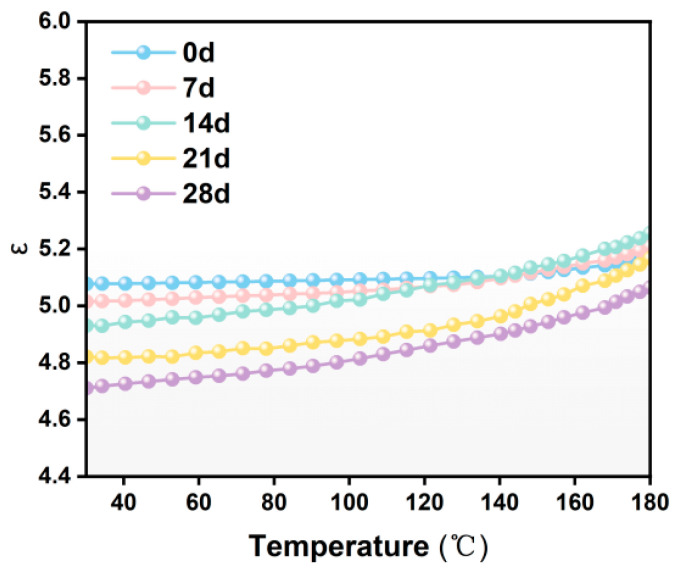
Dielectric constant of Class F insulation materials after different low-temperature treatment durations.

The dielectric constant, a physical quantity describing the degree of polarization of materials under an alternating electric field, is one of the key indicators for evaluating the performance of insulating materials. Figure 4 shows the changes in dielectric constant of F-class insulation materials at −50 °C after 7, 14, 21, and 28 days. The dielectric constant shows a significant increase with the rise in temperature, mainly due to the intensified molecular thermal motion caused by the increase in temperature, which leads to an accelerated response speed of the electric dipole and a shortened relaxation time, thereby promoting the establishment of relaxation polarization. Particularly, when the temperature exceeds approximately 100 °C, the dielectric constant will show a significant increase. This phenomenon is closely related to the glass transition zone (Tg) of the epoxy matrix: below Tg, the molecular chains are in a frozen state, and due to the restricted segment movement, the dipole polarization is restricted; while when the temperature approaches and exceeds Tg, the mobility of the molecular chains significantly increases, allowing the dipole groups to respond more rapidly to the applied electric field and realign, thereby further promoting the polarization process and resulting in a significant increase in the dielectric constant. The dielectric constant gradually decreases with increasing low-temperature treatment time, which is attributed to the accumulation of microcracks and interfacial voids that introduce low-dielectric air regions in the epoxy-mica composite. However, the temperature dependence becomes more pronounced, indicating that low-temperature treatment reduces the thermal stability of the material and shows enhanced interfacial polarization associated with low-temperature-induced structural damage. Although a lower dielectric constant is considered advantageous in some applications, microcracks can become initiation points for partial discharges, further compromising insulation performance and eventually leading to electrical breakdown. Therefore, the presence of microcracks not only affects the electrical integrity of the insulating material but may also shorten the service life of wind turbine stator insulation, increasing the risk of premature failure.

#### 3.2.3. Dielectric Loss Factor of Insulating Materials

**Figure 5:**
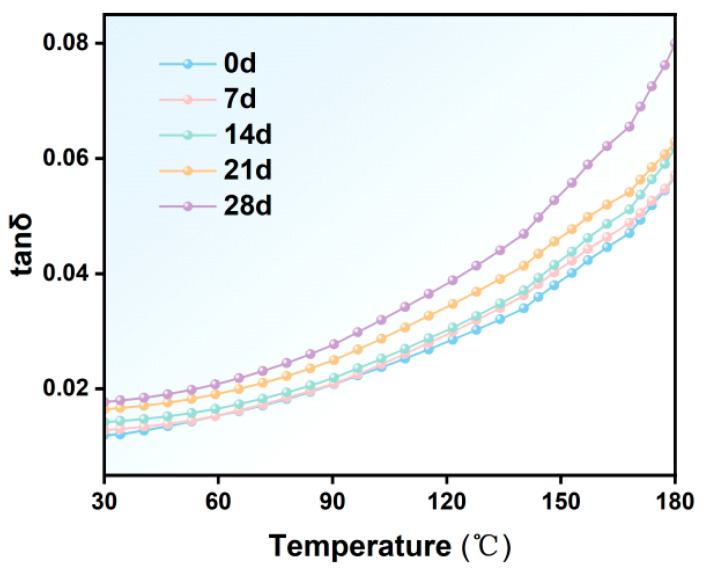
Dielectric loss factor of Class F insulation materials after different low-temperature treatment durations.

Figure 5 shows the changes in the dielectric loss factor of F-class insulation material after 7, 14, 21, and 28 days at −50 °C. The dielectric loss factor gradually increases with extended low-temperature treatment duration, exhibiting a more pronounced change with rising temperature. At 30 °C, tan δ increased from about 0.011 for the untreated sample to about 0.018 after 28 days, and at 180 °C, tan δ increased from about 0.051 to 0.080. This phenomenon indicates that low-temperature treatment not only elevates the baseline dielectric loss but also intensifies polarization and conductive processes. It suggests that as low-temperature treatment duration prolongs, microcracks and interfacial defects gradually accumulate. These microscopic defects enhance energy dissipation pathways. The marked increase in dielectric loss at elevated temperatures further demonstrates that microstructural damage induced by low temperatures amplifies polarization and conductive losses.

#### 3.2.4. Breakdown Electric Field Strength of Insulating Materials

**Figure 6:**
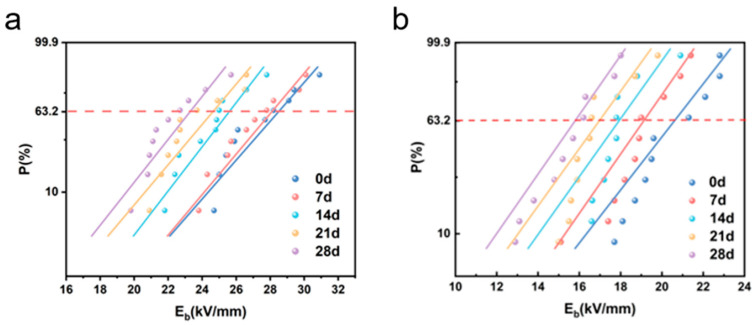
Breakdown field strength of Class F insulation materials after different cryogenic treatment durations. (**a**) The DC breakdown field strength of F-class insulating materials after different durations of low-temperature treatment. (**b**) The AC breakdown field strength of F-class insulating materials after different durations of low-temperature treatment.

Figure 6 Shows the Weibull distribution of the breakdown strength of the epoxy-mica insulation system under different durations of low-temperature exposure: (a) DC breakdown and (b) AC breakdown. The DC breakdown characteristics of the epoxy-mica insulation system exhibit a significant systematic decline with the increase in low-temperature exposure time. The typical breakdown strength decreased from approximately 28.20 kV/mm in the never-aged sample to 22.99 kV/mm after 28 days of exposure, representing a reduction of about 18.5%. This trend is different from the behavior that is often reported in direct low-temperature measurements of pure epoxy resins or molded composite materials, where a sudden drop in temperature can inhibit the movement of the carrier and even increase the breakdown strength [29,30]. The current results indicate that prolonged exposure to low temperatures leads to cumulative degradation rather than instantaneous performance improvement. Compared with the DC results, the alternating current breakdown strength is consistently lower and shows a more significant decrease, reflecting higher sensitivity caused by microstructural defects after prolonged exposure to low temperatures. Additionally, the alternating Weibull curve shows a smaller slope, indicating an increase in statistical dispersion and a decrease in reliability consistency. These results emphasize the crucial role of cumulative microcrack formation and interface degradation in accelerating insulation failure, especially under alternating electric fields. From an engineering perspective, the reduction in characteristic breakdown strength and the increase in dispersion under alternating conditions imply a decrease in insulation safety margin and an increased probability of premature failure in practical applications. From an engineering perspective, a reduction of approximately 18.5% in the DC breakdown strength implies a significant decline in the insulation safety margin. Given that DC breakdown is an important indicator for assessing the inherent integrity of materials, this decrease indicates that the insulation system has become more prone to failure even without considering more complex usage conditions. Therefore, the current research results provide new insights into the long-term reliability of epoxy-cloudier insulation systems under long-term low-temperature storage conditions, emphasizing the necessity of incorporating time-dependent degradation effects in insulation design and evaluation.

### 3.3. Analysis of Degeneration Mechanisms

**Figure 7:**
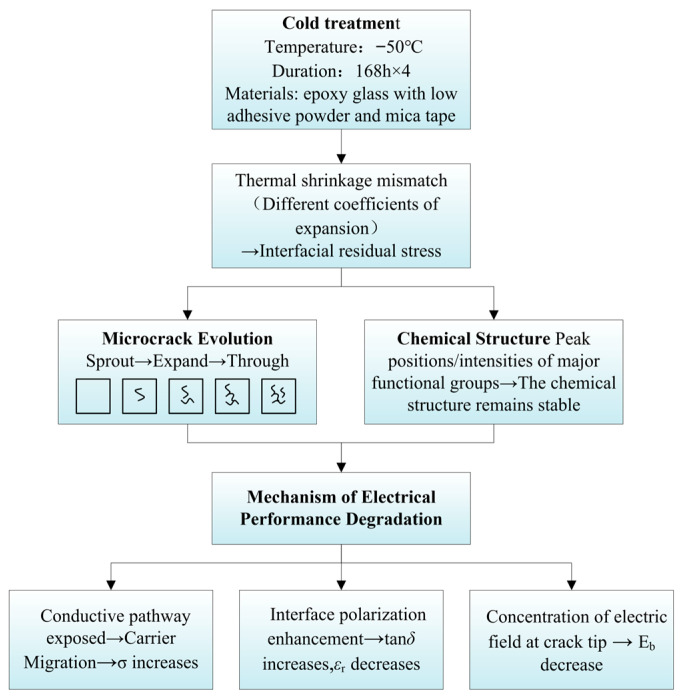
Diagram of Degradation Mechanism for Class F Insulation Materials.

Figure 7 illustrates the mechanism of electrical performance degradation of F-class insulation materials under low-temperature conditions. Based on the analysis results mentioned earlier, it can be concluded that the degradation of F-class mica tapes used in wind turbine stators under low-temperature conditions is not directly caused by a single factor, but rather is a gradual evolutionary process driven by the mismatch of thermal contraction and the expansion of microcracks. Under the −50 °C low-temperature environment, due to the different thermal expansion coefficients among mica paper, epoxy resin matrix, and glass fiber reinforcement materials in the mica tape insulation system, significant thermal contraction mismatch occurs. During the continuous low-temperature action, the contraction degrees of each component are inconsistent, causing the interface area to gradually form thermal stress concentration. Especially between the resin matrix and the mica paper and the glass fiber reinforcement layer, local stress accumulation is more likely to occur. When the interface stress exceeds the local bonding strength, microcracks first appear and interface detachment occurs within the material. The SEM results have shown that after low-temperature treatment, the mica strips have developed various degrees of microcracks, and the interface damage is more obvious. The appearance of microcracks and interface damage will directly destroy the original continuous and dense structure of the mica strips, disrupt the internal current-carrying pathways of the material, and change the migration mode of charge carriers and the interface polarization behavior. It also promotes the accumulation of space charges in local areas, thereby causing an increase in conductivity. The formation of crack areas introduces equivalent air gaps in the local insulating medium, reducing the overall equivalent dielectric constant of the material. On the one hand, the defects reduce the effective volume fraction of the dense medium, and on the other hand, they strengthen the local electric field and the charge migration process, thereby accelerating the formation of the breakdown channel and causing the breakdown field strength to decrease. Under the influence of the low-temperature environment, the microcracks in the insulating material will further expand, and the structural integrity will continue to decline. At the same time, the polarization loss of the material increases, and the thermal conduction path changes, leading to the degradation of its electrical properties. That is to say, the degradation of the insulating material of the mica strip under the low-temperature environment starts with structural damage and is continuously amplified through parameter changes and field responses. As this process continues to develop, the overall reliability of the insulating structure continuously decreases, and eventually may lead to premature aging and even failure of the main insulation of the wind turbine stator.

The prolonged thermoelectric coupling effect eventually led to the electrical decline of the mica tape, showing that “structural damage” gradually evolved into “performance degradation”, and ultimately triggered the aging and failure of the insulation system. This degradation mechanism not only reveals the influence process of the low-temperature environment on the insulation system of wind turbines, but also emphasizes that when designing and selecting insulation materials in cold regions, their low-temperature tolerance and optimized material structure need to be considered, thereby improving the reliability and service life of the insulation system and providing theoretical support for safe and stable operation.

## 4. Conclusions

Based on the analysis of electrical properties and microstructure of typical wind turbine stator main insulation materials after long-term exposure at −50 °C, the following conclusions are drawn:

1. The low-temperature environment of −50 °C will cause micro-cracks and interface damage on the surface of the mica tape. As the duration of the low-temperature treatment increases, the number of cracks gradually increases, and in some local areas, through-cracks and delamination occur. The main reason for this is that the thermal expansion coefficients of the components such as mica paper, resin matrix, and glass fibers are different. Under low-temperature conditions, the contraction degrees of each component are not consistent, resulting in thermal contraction mismatch and stress concentration at the interface, which in turn induces the initiation of micro-cracks and interface detachment. However, the influence of the low-temperature environment on the mica tape essentially manifests as physical structural damage rather than chemical structural destruction.
2. As the duration of the low-temperature treatment increases, the volume conductivity of the mica tape gradually rises, the dielectric loss factor increases, and the power frequency breakdown field strength decreases. Among them, after 28 days of low-temperature treatment, the breakdown field strength of F-grade mica tape decreases by approximately 18.5%. The reason for this is that the microcracks and interface defects formed under low-temperature induction have damaged the original continuous and dense structure of the material, increasing local conductive channels, making charge carriers more likely to migrate, weakening the stability of interface polarization and intensifying energy dissipation, thereby leading to the gradual deterioration of the electrical properties of the insulating material.
3. The low-temperature thermal shrinkage mismatch in the laminated epoxy mica structure leads to cracks, interface gaps, and dielectric discontinuities similar to air gaps. These defects create additional leakage paths, intensify interface polarization, distort the local electric field, and ultimately reduce the reliability of insulation.

This study systematically analyzed the impact of low temperature on the epoxy-zeolite insulation system from a temporal perspective. By combining microscopic structure characterization with macroscopic testing, a correlation between structural damage and performance degradation was established. This degradation mechanism not only reveals the influence process of low temperature environment on the insulation system of wind turbines, but also emphasizes that when designing and selecting insulation materials in cold regions, their low-temperature tolerance and optimized material structure need to be considered, thereby improving the reliability and service life of the insulation system and providing theoretical support for safe and stable operation.

## Figures and Tables

**Table 1:** Sample Composition.

Name	Epoxy Glass Clad Mica Tape
Temperature rating	F-grade (155 °C)
Resin matrix	Epoxy resin
Reinforcing material	Glass fiber cloth
Mica component	Mica paper
Structural feature	Rigid layered composite

## Data Availability

The original contributions presented in this study are included in the article. Further inquiries can be directed to the corresponding author.
